# The Role of Blood Biomarkers for Magnetic Resonance Imaging Diagnosis of Traumatic Brain Injury

**DOI:** 10.3390/medicina56020087

**Published:** 2020-02-22

**Authors:** John K. Yue, Pavan S. Upadhyayula, Lauro N. Avalos, Hansen Deng, Kevin K. W. Wang

**Affiliations:** 1 Department of Neurological Surgery, University of California San Francisco, San Francisco, CA 94143, USA; Lauro.Avalos@ucsf.edu; 2 Brain and Spinal Injury Center, Zuckerberg San Francisco General Hospital, San Francisco, CA 94110, USA; 3 Department of Neurological Surgery, Columbia University Medical Center, New York, NY 10027, USA; pavan8632@gmail.com; 4 Department of Neurological Surgery, University of California Diego, San Diego, CA 92093, USA; 5 Department of Neurological Surgery, University of Pittsburgh Medical Center, Pittsburgh, PA 15213, USA; dengh3@upmc.edu; 6 Brain Rehabilitation Research Center (BRRC), Malcom Randall Veterans Affairs Medical Center, Gainesville, FL 32608, USA; kawangwang17@gmail.com

**Keywords:** blood-based biomarkers, diagnosis, magnetic resonance imaging, traumatic brain injury

## Abstract

*Background and Objectives*: The annual global incidence of traumatic brain injury (TBI) is over 10 million. An estimated 29% of TBI patients with negative computed tomography (CT−) have positive magnetic resonance imaging (MRI+) findings. Judicious use of serum biomarkers with MRI may aid in diagnosis of CT-occult TBI. The current manuscript aimed to evaluate the diagnostic, therapeutic and risk-stratification utility of known biomarkers and intracranial MRI pathology. *Materials and Methods*: The PubMed database was queried with keywords (plasma OR serum) AND (biomarker OR marker OR protein) AND (brain injury/trauma OR head injury/trauma OR concussion) AND (magnetic resonance imaging/MRI) (title/abstract) in English. Seventeen articles on TBI biomarkers and MRI were included: S100 calcium-binding protein B (S100B; *N* = 6), glial fibrillary acidic protein (GFAP; *N* = 3), GFAP/ubiquitin carboxyl-terminal hydrolase-L1 (UCH-L1; *N* = 2), Tau (*N* = 2), neurofilament-light (NF-L; *N* = 2), alpha-synuclein (*N* = 1), and alpha-amino-3-hydroxy-5-methyl-4-isoxazolepropionic acid receptor peptide (AMPAR; *N* = 1). *Results*: Acute GFAP distinguished CT−/MRI+ from CT−/MRI− (AUC = 0.777, 0.852 at 9–16 h). GFAP discriminated CT−/diffuse axonal injury (DAI+) from controls (AUC = 0.903). Tau correlated directly with number of head strikes and inversely with white matter fractional anisotropy (FA), and a cutoff > 1.5 pg/mL discriminated between DAI+ and DAI− (sensitivity = 74%/specificity = 69%). NF-L had 100% discrimination of DAI in severe TBI and correlated with FA. Low alpha-synuclein was associated with poorer functional connectivity. AMPAR cutoff > 0.4 ng/mL had a sensitivity of 91% and a specificity of 92% for concussion and was associated with minor MRI findings. Low/undetectable S100B had a high negative predictive value for CT/MRI pathology. UCH-L1 showed no notable correlations with MRI. *Conclusions*: An acute circulating biomarker capable of discriminating intracranial MRI abnormalities is critical to establishing diagnosis for CT-occult TBI and can triage patients who may benefit from outpatient MRI, surveillance and/or follow up with TBI specialists. GFAP has shown diagnostic potential for MRI findings such as DAI and awaits further validation. Tau shows promise in detecting DAI and disrupted functional connectivity. Candidate biomarkers should be evaluated within the context of analytical performance of the assays used, as well as the post-injury timeframe for blood collection relative to MRI abnormalities.

## 1. Introduction

Traumatic brain injury (TBI) is a leading cause of death and disability, with an annual global incidence of over 10 million cases. Accurate diagnosis, triage and treatment are critical public health necessities [1,2,3]. Initial evaluation utilizes the Glasgow Coma Scale (GCS) score, which incorporates eye, verbal, and motor response to categorize neurologic dysfunction into severe (GCS 3–8), moderate (GCS 9–12) and mild (GCS 13–15) TBI. It is increasingly evident that even patients with mild TBI (mTBI) are at risk of intracranial hemorrhage and non-grossly hemorrhagic injury such as axonal shear on presentation, as well as persistent functional deficits at or beyond 3 months post-injury [4]. Evidence over the past two decades has shown that GCS alone may not be sufficient to diagnose or prognosticate TBI, especially on the mild end of the spectrum [3].

Current standard of care for TBI relies on computed tomography (CT) due to its specificity for hemorrhage, fractures, and rapid acquisition time [5]. However, magnetic resonance imaging (MRI) has been shown to be increasingly important for certain TBI subpopulations. MRI is more sensitive to nearly all forms of intracranial injury apart from acute hemorrhage and fractures due to its ability for evaluation of axonal shear, small contusions, and hypoxic injury. Beyond its use in the acute TBI, MRI can also evaluate volumetric loss in chronic TBI. Acute TBI may present as CT negative/MRI positive (CT−/MRI+) or CT negative/MRI negative (CT−/MRI−). The use of adjunct diagnostic techniques such as serum biomarkers can further diagnose and/or risk stratify patients with suspected TBI. The ideal serum biomarker should have high specificity for brain tissue, high sensitivity for brain injury, and stable kinetics. Several serum biomarkers have been widely studied in clinical settings as potential biomarkers for TBI severity and risk stratification. These include S100 calcium-binding protein B (S100B), glial fibrillary acidic protein (GFAP), ubiquitin carboxy-terminal hydrolase-L1 (UCH-L1), neurofilament light protein (NF-L), Tau protein, and others. We summarize the relevant data related to these biomarkers and their utility for diagnosis of TBI.

## 2. Materials and Methods

### 2.1. Study Selection

The National Library of Medicine PubMed database was queried using the search terms ((plasma (title/abstract) OR serum (title/abstract)) AND (biomarker (title/abstract) OR marker (title/abstract) OR protein (title/abstract)) AND (brain injury (title/abstract) OR head injury (title/abstract) OR head trauma (title/abstract) OR concussion (title/abstract)) AND (magnetic resonance imaging (title/abstract) OR MRI (title/abstract)) in the English language. Primary literature on humans, excluding case reports, was included in the current review. Study authors reviewed each article and determined its relevance to plasma/serum biomarkers and MRI (magnetic resonance imaging) in TBI, and all articles were unanimously included or excluded. 

### 2.2. Inclusion and Exclusion Criteria

Studies were included if they studied an adult population (age > 18), studied a patient population with traumatic brain injury, studied a serum biomarker and had patients who had MRI data. Studies were excluded if they were not in the English language, were animal research or basic science, lacked MRI imaging, or were case reports or reviews. 

### 2.3. Article Summary

**Figure 1:**
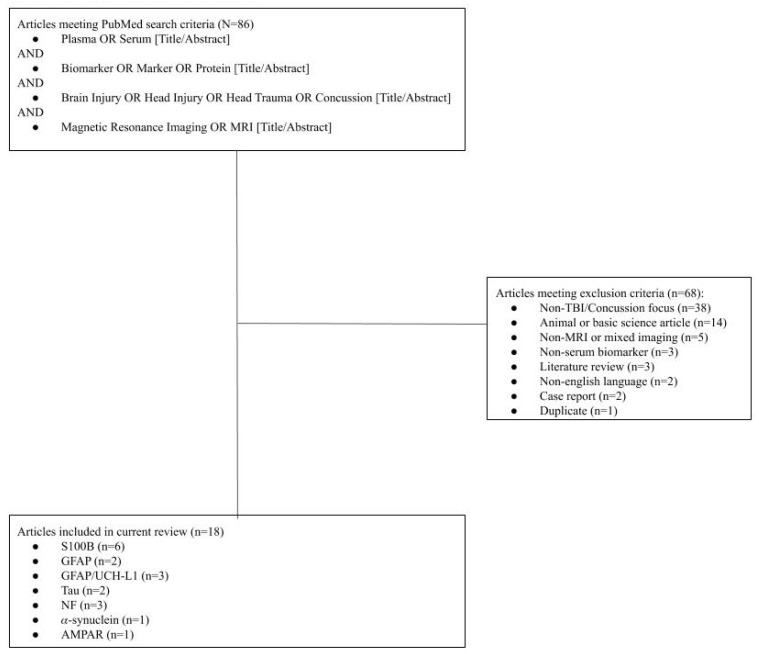
Flow Diagram of Included Articles.

Of 86 articles from the initial search, 18 were selected for inclusion (Figure 1), which included S100B (*N* = 6), GFAP (*N* = 3), GFAP/UCH-L1 (*N* = 2), Tau (*N* = 2), NF-L (*N* = 2), and alpha-synuclein (*N* = 1), AMPAR (*N* = 1). Sixty-eight articles were excluded for the following reasons: article was in non-English language (*N* = 2), animal or basic science focus (*N* = 14), the primary focus was not TBI/concussion (*N* = 38), no or mixed MRI data (*N* = 5), non-serum biomarker focus (*N* = 3) case report (*N* = 2), literature review (*N* = 3), and duplicate entry (*N* = 1).

## 3. Results

Table 1 displays studies qualifying for review in the current manuscript. 

### 3.1. S100 Calcium-Binding Protein B (S100B; 6 Studies)

S100B is a calcium-binding protein synthesized in astroglial and Schwann cells, primarily found in white matter, and has been one of the longest-studied proteomic biomarkers for TBI [6]. S100B has a half-life of 2 h. The earliest prospective study on S100B and radiographic evidence was performed by Herrmann et al. in 1999. This study, however, did not differentiate between CT and MRI, nor did they find associations between S100B release patterns and radiographic findings [6]. Ingebrigtsen et al., studied 45 CT-negative mTBI patients who received 0.5 T MRIs and had S100B drawn immediately at presentation as well as at a second time point within 12 h of presentation. Mean S100B declined from time of injury, and within the 28% with detectable S100B, 60% had returned to below the detection limit within 7 h of injury. Four of the five patients (80%) were MRI+ for contusion and had elevated serum S100B (>0.2 ug/L), which differed significantly with those who were MRI-negative. Patients with elevated S100B also had poorer 3 month attention, memory, and processing speed [7]. Romner et al. drew S100B within 24 h of injury in mTBI patients—a subset of whom underwent 0.5 T MRI (*N* = 45) and five had contusions. While not a direct comparison, 80% of those with MRI+ contusions had S100B >0.2 ug/L, vs. 30% for those who were radiographically negative (*N* = 228) [8]. The authors concluded that undetectable S100B predicts normal intracranial findings on CT, although no comment is made for MRI [8]. In 2007, Oh et al. investigated the Elecsys immunoassay in the ED in 32 TBI patients who were CT/MRI+ (mean 0.253 ug/L) vs. 13 patients who were CT−/MRI− (mean 0.098 ug/L, *p* = 0.028) [9]. The authors found a similar trend in nontraumatic brain injury (0.179 vs. 0.057 ug/L; *N* = 34 vs. *N* = 22, *p* = 0.037). The overall AUC for detecting CT−/MRI+ vs. CT−/MRI− brain injury was 0.839 (95% CI 0.748–0.929, *p* < 0.001), with a sensitivity of 84.8% and specificity of 74.3% at a S100B cutoff of 0.105 ug/L. However, the authors do not differentiate between CT and MRI+ brain injury [9]. In 2013, Thelin et al. reported on 250 TBI patients regarding a second S100B peak after 48 h of injury in 39% of patients, which correlated with radiographic injury – most frequently ischemia/infarction (70 of 98) followed by edema (15 of 98) [10]. This study, similar to other aforementioned studies, did not differentiate between CT and MRI findings and therefore is of limited utility to the goals of the current review.

Finally, a small study in 2016 by Linsenmaier et al. using the Elecsys S100B assay showed in 41 mTBI patients that while all 29 CT-negative scans were 1.5 T MRI-negative, of the 12 CT+ scans, only five were MRI+; six were false positives, and one was a cavernous angioma (nontraumatic) [11]. Using a S100B cutoff of 0.1 ug/L, the study found PPV 16% and NPV 100% for MRI+ intracranial lesions [11].

In 2013, Marchi et al. investigated pre- and post-game (<24 h) serum S100B levels in 67 non-concussed college football players and obtained three diffusion tensor imaging (DTI) scans to evaluate for white matter changes [12]. The authors found that transient blood–brain barrier (BBB) damage measured by S100B, as well as S100B auto-antibodies, was detected in players with the greatest number of subconcussive head strikes (6 strikes: mean 0.10 ug/L, 0 strikes: mean 0.02 ug/L, *p* = 0.030; played with head hits: mean 0.11 ug/L, played <5 min: mean 0.01 ug/L) [12]. S100B auto-antibodies also predicted persistent DTI abnormalities and cognitive changes. The authors also show that in the absence of concussions, S100B tends to return quickly to baseline after a game. The authors concluded that cumulative subconcussive head strikes may lead to BBB disruption, and elevated serum S100B, with implications for risk assessment [12]. There is also the implication that circulating autoantibodies against a CNS antigen can become pathogenic to the brain when BBB disruption occurs and/or otherwise allows their access into the brain [13].

### 3.2. Glial Fibrillary Acidic Protein (GFAP; 5 Studies)

GFAP is an intermediate filament abundantly present in astrocytes of the central nervous system. The reactive astrocytic response to brain trauma in the context of BBB disruption forms the basis of interest in this biomarker of TBI [14]. The literature on the relationship between GFAP and MRI in TBI has been mixed. In 2017 Posti et al. compared 93 patients with mTBI to 73 patients with acute orthopedic trauma. Serum was collected on days 1, 2, 3, and 7 after admission. GFAP breakdown products (GFAP-BDPs) were measured using proteomic analyses from Randox Laboratories based on horseradish peroxidase chemiluminescent detection. The authors noted that GFAP was higher in the orthopedic trauma compared to CT-negative mTBI patients on day 1 (*p* = 0.026), with no differences on subsequent days [15]. Only patients in the orthopedic control group received MRIs, with no correlation being found between abnormal MRI pathology and GFAP serum levels. MRIs were comprehensive including DTI, high-resolution 3D T1 and normal T1/T2 parameters to ensure pathology was not missed. Understandably, this has limited application to the mTBI cohort [15].

In contrast, a small descriptive study by Kou et al. examined nine ED mTBI patients who received blood draws every 6 h for the first 24 h post-injury. Mean serum GFAP for mTBI was 10.6 times higher than controls at admission (0.043 ng/mL vs. 0.004 ng/mL; *p* < 0.01). Multiple imaging modalities were used to determine MRI pathology including DTI. GFAP levels were much higher in patients with hemorrhagic findings on MRI (mean 0.517 ng/mL). The authors even describe one case where a small intraventricular hemorrhage near the sublingual gyrus was missed on CT but present on MRI and was associated with a marked increase in GFAP levels that persisted across the 24 h sampling period (median 4.61 ng/mL). However, DTI findings did not correlate with GFAP levels [16]. The Transforming Research and Clinical Knowledge in Traumatic Brain Injury (TRACK-TBI) Pilot analysis by McMahon et al. showed the utility of GFAP breakdown products (GFAP-BDPs) for stratification of TBI severity. In 215 patients (mild TBI 83%, moderate TBI 4%, severe TBI 12%), GFAP-BDP levels were associated with increased injury severity on CT as classified by the Rotterdam Score in both univariate (OR 1.2; *p* < 0.001) and multivariate (OR 1.13; *p* < 0.001) analyses [17]. GFAP-BDP values at admission were significantly higher in MRI-positive patients (1.31 ± 1.8 vs. 0.28 ± 0.57 ng/mL; *p* < 0.001). GFAP-BDP levels also predicted the presence of MRI pathology on both univariate (*OR* = 2.7) and multivariate (*OR* = 3.8) analyses. However, no differences in GFAP-BDPs were seen between CT−/MRI+ and CT−/MRI− patients [17]. Gill et al. analyzed serum GFAP, tau and NF-L in mTBI patients with CT and MRI. GFAP had AUC = 0.93 in distinguishing the 277 patients with mTBI from 49 healthy controls on CT and was the only marker able to stratify mTBI patients to CT+ vs. CT− findings. GFAP was also the strongest predictor for CT−/MRI+ findings compared to healthy controls with AUC = 0.803 and a significant difference between the different patient cohorts (healthy controls: 56.18 pg/mL (45.98–69.84), CT+/MRI+: 2853 pg/mL (768.2–5724), CT−/MRI+: 2098 pg/mL (253.7–8382), CT−/MRI−: 266.2 pg/mL (101.8–857.2); *p* < 0.001) [18]. Importantly, these authors used multimodal MRI with high-resolution 3D-T1 images, which may be more sensitive than conventional MR techniques.

In the largest study in CT− mTBI with MRIs to date, Yue et al. demonstrated the relationship between GFAP and CT−/MRI+ patients compared to CT−/MRI−, orthopedic controls, and healthy controls using the 18 center prospective TRACK-TBI study in 2019 [19]. Serum from patients was drawn within 24 h of TBI and correlated with brain MRI 7–18 days post-injury. A prototype point-of-care immunoassay, capable of generating results within 15 minutes in a controlled setting, was performed at Abbott Laboratories. CT−/MRI+ patients had significantly higher serum GFAP (414.4 pg/mL [IQR 139.3–813.4]) compared to CT−/MRI− (74.0 pg/mL [17.5–214.4]), orthopedic controls (13.1 pg/mL [6.9–20.0]) and healthy controls (8.0 pg/mL [3.0–14.0]). AUC of GFAP for detecting MRI abnormalities in CT− patients ranged from 0.719–0.852 within the first 24 h and was most sensitive between 9–16 h (AUC 0.852) [19]. Furthermore, GFAP was found to be most sensitive for detecting diffuse axonal injury (DAI) on MRI, with AUC 0.903 for distinguishing DAI+ from CT-/MRI- patients, and AUC 0.976 for distinguishing DAI+ from orthopedic controls. The ability to use GFAP to reliably determine the presence of MRI pathology will allow patients who could benefit from advanced imaging to have a higher likelihood of receiving it. Using GFAP in risk stratification and decision-making for targeted intervention can save patients from unnecessary scans while also maximizing the utility of healthcare resources.

### 3.3. Ubiquitin Carboxy-Terminal Hydrolase L1 (UCH-L1; 2 Studies)

UCH-L1 is a protease enzyme that comprises up to 10% of all neuronal proteins [20]. UCH-L1 can be detected in serum in hours after severe TBI [21]. Two studies that examined the role of plasma GFAP levels also studied plasma UCH-L1, likely related to their different cell of origin. UCH-L1 is a neuronal protein; studies examining the ratio of glial:neuronal proteins (e.g., GFAP:UCH-L1) have shown that this glial:neuronal ratio correlated with the presence of traumatic lesions on CT but not with diffuse injury [20]. For example, Gill et al. measured GFAP and UCH-L1 simultaneously using a Simoa multiplex assay (Quanterix, MA) [18]. While UCH-L1 was unable to discriminate between CT−/MRI+ and CT−/MRI−, the authors state that UCH-L1 measurements did not meet internal quality control standards and that these data require further study. Kou et al. reported that UCH-L1 after mTBI was significantly elevated compared to healthy controls (0.242 vs. 0.05 ng/mL; *p* < 0.01). However, this did not correlate with the number of MRI lesions [16]. These authors also used a 3 T magnet with tractography so their findings should be sensitive for MR pathology [16]. While UCH-L1 does not seem to be sensitive for MR pathology, its role as a neuronal protein could still be used in a multimodal serum-based risk stratification system given its known increase after TBI and correlation with CT pathologies.

### 3.4. Neurofilament Proteins (NF-L; 3 Studies)

Neurofilament light (NF-L) protein is a component of axonal intermediate filaments thought to be released following axonal injury. In contrast to other biomarkers, it has a long half-life in vivo of 3 weeks [22], and thus may be of use in TBI patients with delayed or subacute presentation. In a small case series of nine severe TBI patients with DAI, Ljungqvist et al. showed that serum NF-L, measured using a Simoa assay (Quanterix, MA), was 30-fold higher in severe TBI compared to controls with 100% discrimination [23]. High NF-L correlated with low fractional anisotropy (*R*^2^ = 0.83)—as measured on a 1.5 T MRI by diffusion tensor imaging—and high average diffusivity on DTI (*R*^2^ = 0.79) [23]. A second study by Al Nimer et al. evaluated the relationship between NF-L and DAI in 182 patients (mTBI = 8%, moderate TBI = 21%, severe TBI = 71%) [24]. NF-L levels remained relatively constant from admission to 2 weeks post-injury. A multivariable proportional odds model found that the addition of NF-L to core predictors (age, GCS and pupillary response) improved Nagelkerke pseudo-*R*^2^ from 0.25 to 0.30 for prediction of outcome based on the Glasgow Outcome Scale (GOS) [24]. No correlation between DAI, as measured by echo planar diffusion MRI, and NF-L levels was noted. Importantly, both studies were skewed towards severe TBI, and understanding whether NF-L can discriminate between MRI findings in the mTBI population remains in need of further study.

A third study by Sandsmark et al. looked at neurofilament heavy chain (NF-H) in 76 traumatic brain injury patients. MRI was used to separate patients into those with traumatic microvascular injury (TVI) defined as diffusion weighted MRI without frank hemorrhage, those with frank hemorrhage (TH) and those with no injury on MRI. NF-H was increased in both TH and TVI groups compared to MRI− group within the first 48 h following injury [25]. Importantly, NF-H plasma levels remained elevated after 48 h in the TVI group but not the TH group. These findings show that neurofilament proteins can be a marker for DAI and TVI, and will require future confirmation studies in the larger mTBI population.

### 3.5. Tau Protein (2 Studies)

Tau is a tubulin-associated protein necessary for microtubule polymerization, with numerous physiologic functions including regulation of synaptic transmission and cell cycle regulation. Phosphorylation allows for depolymerization of the tubule bundles, and phosphorylated tau has been linked with various neuropathologic conditions including TBI and Alzheimer’s Disease [26]. Recently, Hirad et al. examined the effects of repetitive subconcussive head impacts (RSHIs) by comparing 29 patients with clinically defined mTBI, 38 collegiate football players with helmet-based accelerometers, and 58 healthy controls. The authors hypothesized that both RSHI mTBI patients would have pathologic white matter changes consistent with DAI. DAI was measured using diffusion tensor imaging on a 3 T magnet in a collegiate football cohort. Traumatic strikes with high rotational acceleration was inversely correlated with fractional anisotropy in midbrain white matter (*r* = −0.43, *p* < 0.008). The number of hits with high linear acceleration was also correlated (*r* = −0.32, *p* < 0.049). In the mTBI cohort, a similar change in fractional anisotropy in the midbrain was also seen. In 13 of 29 subjects in the mTBI cohort, blood was collected within 72 h of injury and serum tau was inversely correlated with midbrain fractional anisotropy (*r* = −0.60, *p* < 0.033) [27].

Tomita et al. examined serum tau in 40 TBI patients grouped into two cohorts using MRI: DAI+ (*n* = 13) and DAI− (*n* = 27). DAI+ patients had significantly higher serum tau (25.3 vs. 0.0 pg/mL, *p* = 0.03) and the receiver operating statistic of serum tau at 1.5 pg/mL showed good sensitivity (74.1%) and specificity (69.2%) for diagnosis of DAI on diffusion-weighted imaging (DWI). Unfortunately, plasma tau was unable to predict unfavorable outcome (GOS) (*p* = 0.19) [28]. The ability for tau to correlate with fractional anisotropy changes and DAI reflect its promise as a serum biomarker; standardized MR techniques may help further improve the prognostic utility of tau protein.

### 3.6. Alpha-Synuclein (α-Synuclein; 1 Study)

Beyond the classification of axonal versus neuronal injury, specific biomarkers may allow for identification of altered neural connectivity. α-Synuclein is a presynaptic neuronal protein seen in various forms of dementia. In 2019 Ye et al. examined serum α-synuclein in 52 mTBI patients and 47 healthy controls using a multiplex assay (Luminex Corp, Austin, TX, USA) [29]. Patients were evaluated with functional MRI and comprehensive neuropsychological testing. MTBI patients were divided into high and low α-Synuclein groups based on biomarker levels in healthy controls. Low α-synuclein levels were associated with higher post-concussional (PCS; *β* = −0.333, *p* = 0.013) and depression symptoms (*β* = −0.311, *p* = 0.022). In default mode network studies, higher α-synuclein correlated with greater functional connectivity in the left anterior cingulate and ventral medial prefrontal cortices (*p* < 0.05). As α-synuclein is a presynaptic neuronal protein present in dementia, this finding may seem paradoxical. The authors suggest that the higher α-synuclein levels in patients with greater connectivity and better outcomes may be due to a compensatory mechanism whereas lower levels are due to lack of compensation [29].

### 3.7. Alpha-Amino-3-Hydroxy-5-Methyl-4-Isoxazolepropionic Acid Receptor Peptide (AMPAR; 1 Study)

AMPAR is a product of the proteolytic degradation of AMPA receptors, which provide glutamatergic synaptic transmission. Neural networks can develop excitotoxic activity in TBI, resulting in large glutamate release and AMPAR upregulation [30,31,32]. Disturbances in glutamate synaptic transmission during concussion may be measurable with plasma AMPAR. In 2013, Dambinova et al. studied 84 athletes and 40 nonathletes pre- and post-collegiate sport season [33]. AMPAR was analyzed using magnetic antibody precipitation with light absorbance quantification. Outcomes were assessed using the ImPACT neurocognitive battery. Thirty-three athletes had concussions post-season and were age- and gender-matched to the other 91 controls without concussion. In general, the concussed group had higher AMPAR (2.15 ng/mL (0.96–8.49)) vs. nonathletes (0.19 ng/mL (0.04–0.40)). However, three subjects with concussion had low AMPAR (0.20–0.36 ng/mL) and seven non-athletes had elevated AMPAR (0.44–1.10 ng/mL). At a cutoff of 0.4 ng/mL, AMPAR had a sensitivity of 91% and specificity of 92% for detecting concussions. The three subjects who received MRIs had multiple concussions, high AMPAR, and decreased ImPACT scores. MRI included T1 and T2 FLAIR images which may be less sensitive to axonal shear than diffusion weighted imaging. Of these, two had resolution of symptoms over 3 weeks, with declining AMPAR levels, without evidence of axonal shear on MRI. The third patient continued to have high AMPAR levels (5.2 ng/mL) 6 months post-injury, with spots of microhemorrhage in deep brain structures and changes in high convexity white matter [33]. This study highlights the utility of drawing longitudinal AMPAR levels as it may correlate with persistent TBI pathology.

## 4. Discussion

In the U.S. alone, 1.7–2.0 million TBI cases are documented annually with mTBI cases comprising over 85% of total cases [34,35]. The overall incidence is likely much higher due to patients who do not reach care and/or are not included in national statistics. Diagnosis of TBI presents a unique clinical challenge as the disease presents heterogeneously, and alterations in consciousness can be due to a number of pathophysiological mechanisms. Proper treatment of TBI relies upon proper diagnosis and clear understanding of intracranial pathology.

### 4.1. Relevance of MRI-Based TBI Diagnosis and Rationale for Serum Biomarker Use

Although MRI is more sensitive than CT for detecting changes in virtually all post-traumatic lesions except for acute fractures or hemorrhage, the ability to obtain routine MRIs is limited by resource availability and cost. For this reason, the American College of Emergency Physicians (ACEP)/Centers for Disease Control (CDC) guidelines for mTBI do not recommend MRI for the evaluation of acute TBI [5]. Recent data, however, show that nearly 29% of mTBI are CT-occult [36]. This constitutes one reason that over time, mTBI patients who have similar acute presentations can either achieve full functional recovery or can have persistent neuropsychiatric sequelae and functional deficits [36].

While MRI may help direct resources to this undertreated patient population, a few challenges do exist. Namely standardization due to different magnets, acquisition parameters, and processing algorithms means the generalizability of MRI findings may be limited [37,38]. While MRI can help direct resources to this patient population; standardized guidelines for MRI acquisition are needed to ensure that data between sites are readily comparable. 

The importance of MRI findings, especially in mTBI patients with normal CT, was highlighted by Yuh et al. in 2013. MRI pathology in the form of contusions or DAI in CT-negative mTBI were independently associated with poorer 3 month functional outcomes on GOSE [36]. Based on CT scans alone, these patients would be considered “uninjured” with few to no standard follow-up instructions [39]. High rates of post-traumatic stress disorder and major depressive disorder following mTBI are associated with CT-occult MRI findings [40]. This further highlights the need for targeted utilization of MRI for determining which mTBI patients are at elevated risk and who should be prioritized for healthcare surveillance and follow up [41,42].

Use of a circulating biomarker capable of diagnosing CT-occult mTBI could significantly improve TBI diagnosis. The smaller retrospective studies reported in this review have few MRIs (~9%) [33]. These low rates even in clinical trials, such as in TRACK-TBI Pilot (~40%), show the barriers to access for MRI [43]. Cost-effectiveness data for the use of a biomarker for screening show that a biomarker assay should cost less than $308.96 to be more effective than standard CT in TBI patients [44]. It stands to reason that the cost-effectiveness cutoff for MRI is much higher. Comparatively, a study in Sweden showed the cost of serum sampling of S100B is only 21 euros [45]. Together, these data show the potential for both cost savings and improved diagnostic accuracy.

Biomarker studies have had marked success in discriminating intracranial findings on CT with AUCs of > 0.90. This success led to U.S. Food and Drug Administration (FDA) clearance of a composite biomarker assay measuring acute GFAP and UCHL-1 levels in blood for differentiation of patients with normal versus abnormal CT including the pivotal clinical trial ALERT-TBI with 1977 TBI patients with initial GCS 9–15 [46,47,48,49]. Serum S100B has been incorporated into Scandinavian TBI guidelines in triaging mTBI patients who may need CT [50]. Unfortunately, validation of biomarkers for MRI remains a challenge largely due to the large cost associated with MRI. Moreover, since MRI is not part of the current standard of care clinical TBI guidelines, studies to date remain sparse [5]. The final hurdle with using serum biomarkers to stratify MRI findings is due to the various temporal cascades of different serum biomarkers, related to cell type of origin and inflammatory responses. Acute biomarkers, e.g., neuronal/axonal injury markers (UCH-L1), blood–brain barrier breakdown markers (S100B) and reactive gliosis or glial injury markers (ie. GFAP), peak within hours of injury. Subacute markers, such as NF-L may peak within days to weeks. Chronic biomarkers such as Tau/phosphorylated Tau may peak in weeks to months and signify neurodegenerative changes [51]. These data show the difficulties in defining an optimal timeframe and serum biomarker combination that can be of use in stratifying TBI patients according to MRI pathology.

This challenge is best demonstrated by the diagnostic biomarker S100B. The data assessing the utility of S100B for diagnosis are mixed. S100B levels are shown to return to baseline within 7 h of injury, creating a short window for serum acquisition [7,8]. This fact may underlie the heterogeneity in the published relationships between S100B levels and neuroimaging findings. Previous studies have failed to link BBB dysfunction (assessed via dynamic contrast MRI) with serum S100B levels [52]. Studies by Romner, Oh, Linsemaier, and Ingebritsen all show that low/undetectable serum S100B (<0.2 ug/L) has high negative predictive value for neuroimaging pathology, and thus rarely have true pathologic findings on CT or MRI [7,8,9,11]. This finding shows S100B’s utility in preventing unnecessary CTs, but underscores its limitation in determining MRI pathology.

### 4.2. GFAP Has High Diagnostic Potential for MRI

Of the biomarkers currently under consideration, the one with the greatest potential is GFAP. GFAP has already received FDA approval to evaluate intracranial injury given the high predictive capacity for CT findings [17]. The 18 center TRACK-TBI study with interim analysis of 450 CT− mTBI patients with MRIs, 122 orthopedic controls and 209 healthy controls serves as preliminary Phase III evidence of the ability for GFAP to distinguish between CT−/MRI+ and CT−/MRI− TBI [19]. Although still awaiting validation from the full TRACK-TBI cohort, Yue et al. show that GFAP collected within 24 h post-injury had AUC of 0.777 for discriminating CT−/MRI+ from CT−/MRI−, which rises to 0.852 in the 9–16 h cohort [19]. Perhaps even more striking, subgroup analysis showed that GFAP was able to discriminate DAI+ from CT−/MRI− patients with AUC = 0.903 [19]. These data are supported by prior smaller studies, including Gill et al. which showed that GFAP-BDPs could be used to discriminate between CT−/MRI+ and healthy controls, and by Kou et al. which showed increased GFAP levels in CT−/MRI+ patients with intraventricular hemorrhage [16,18]. These findings show the promise of serum GFAP as a diagnostic TBI biomarker, and in evaluating suspicion for specific intracranial pathologies on MRI.

### 4.3. Tau Has Possible Diagnostic Potential for MRI

Tau also shows promise as a serum biomarker of intracranial pathology on MRI. As a marker of neurodegeneration, Tau persists in circulation for weeks to months following TBI [28]. For this reason, serum Tao monitoring can enable serial analyses with post-acute MRIs for continued follow up. Tomita et al. showed that Tau is elevated in cases of DAI and has a sensitivity of 74.1 for diagnosing DAI [28]. Hirad et al. showed that high serum Tau correlated with lower fractional anisotropy and hence disrupted white matter [27]. Moreover, Tau levels correlated with the number of strikes in concussed athletes, and thus may be a potential marker that is dose-dependent and congruent with the clinical reality of multiple impacts [27]. Early studies from the TRACK-TBI pilot cohort have also shown that phospho-Tau shows good discriminability for chronic TBI versus healthy controls with an AUC of 0.963–1.00 [53].

### 4.4. Biomarkers in Need of Further Research for Diagnostic Potential on MRI

NF-L has potential for risk stratification beyond the acute injury window. As it persists for up to 3 weeks in serum, it provides a subacute measure that may correlate with both MRI and long term outcomes. While Ljungvist et al. showed that high NF-L levels correlated with lower fractional anisotropy, their study was small (limited to nine patients) [23]. Other larger studies, such as the one by Al Nimer have not demonstrated similar findings showing no correlation between DAI and NF-L levels. Although the data are mixed, the ability for a biomarker to predict MRI changes in the subacute or chronic phase following TBI warrants further investigation [24].

Two emerging markers for MRI abnormalities remain in need of larger confirmatory studies. α-Synuclein may be a marker for disrupted connectivity and reduced fractional anisotropy. Ye et al. showed that in TBI patients, high α-synuclein is associated with greater functional connectivity; this paradoxical finding may be related to a compensatory mechanism following brain injury that utilizes this presynaptic neuronal protein. Importantly, the low levels of α-synuclein not only predicted decreased functional connectivity, but also greater post-concussional symptoms [29]. This shows that α-synuclein may be important for an outcomes-based treatment approach. The second marker of interest is AMPAR, a marker of glutamatergic dysregulation. While patients with multiple concussions and increased symptomatology also had increased circulating AMPAR, only one had verifiable MRI abnormality [33]. These studies parallel to results from GFAP, where CT−/MRI− TBI had higher circulating GFAP (mean 74 pg/mL) compared to orthopedic (13 pg/mL) and healthy controls (8 pg/mL), suggesting that even CT−/MRI− patients may have subclinical damage to the brain after TBI [19].

## 5. Limitations

TBI studies to date on biomarkers and MRI remain low in number and sample size and are often secondary endpoints to CT-based studies, thus constituting mostly Level III/IV evidence. Thresholds for detection of MRI abnormalities must be established for biomarkers of interest, with GFAP at the forefront in near-term potential for diagnosis [19]. A key factor beyond control in this review was the different imaging parameters in different studies. Since detection of MRI abnormalities depends on magnet strength, imaging parameters and post-processing, standardization of imaging parameters through consensus protocols can help improve standardization of the serum biomarker findings across larger patient populations. Many serum biomarkers correlated with axonal injury and the detectability of axonal injury dramatically differs whether structural MRI or advanced tractography is being used. This highlights the need for further large-scale multi-institution studies for the evaluation of the utility of diagnostic serum biomarkers in MRI.

Relevant temporal cascades of biomarkers of interest also need to be established with MRIs. In the current research environment, MRIs are often acquired much later than the initial blood draw and thus generalizability is limited. However, this argues for the case of using a sensitive biomarker for triage to subacute or outpatient MRI after mTBI. Furthermore, in most studies to date, biosamples were collected with different post-injury times and their results aggregated. As different biomarkers have different half-lives in circulation, biomarker values and their correlation with imaging may differ depending on time of draw. As stated earlier, MRI is not currently standard of care for TBI [5]. However, given the recent evidence on biomarker correlates of CT-occult injuries, the next TBI guidelines may benefit from inclusion of MRI in targeted mTBI subpopulations. While out of scope of the current paper, relevant biomarkers for prognosis after TBI constitutes another imminent future direction.

## 6. Conclusions

Under the current standard of care, TBI cases without CT abnormalities are discharged with return precautions but may have subclinical injuries and persistent functional symptoms. An acute circulating biomarker able to discriminate MRI abnormalities will be critical to the diagnosis of CT-occult TBI. MRI in trauma and acute care is a limited resource and a biomarker with high discrimination for MRI+ intracranial injury can triage patients who may benefit from outpatient MRI, increased surveillance and/or post-acute follow up with a TBI specialist. Of current markers in literature, the glial marker GFAP has shown high potential for diagnostic efficacy for MRI+ intracranial injury (notably for DAI), and awaits Phase III validation studies. The neurodegenerative marker Tau shows promise in detecting DAI and disrupted functional connectivity. Thresholds for detection of MRI abnormalities, and considerations for temporal cascades in their release after blood brain barrier disruption, must be established for biomarkers of interest.

## Figures and Tables

**Table 1:** Summary of Included Studies.

***S100B***							
**Author and Year**	**Study Type**	***N***	**MRI Acquisition Parameters**	**Description**	**Outcome Measures**	**Timing of Serum Sampling**	**Results**
Ingebrigtsen et al., 1999	Prospective Cohort	50 TBI	0.5 T magnet; T1/T2 FLAIR	Validation of S100 as a serum biomarker for brain injury.	Serum S100B	On admission and 12 h post-injury	In total, 14/50 (28%) of patients had detected serum S100 (mean 0.4 ug/L ± 0.3), with levels being highest immediately after injury and declining each hour after. S100B not detectable in 36% of patients after 6 h of initial detection. There were five MRI+ patients with brain contusions—of which have had elevated S100B.
Linsenmaier et al., 2016	Prospective Cohort	41 TBI	1.5 T magnet; T1/T2; GRE; DWI	Feasibility study of S100B as biomarker in mTBI with MRI.	Serum S100B CT; MRI	Hourly, until 12 h post-injury	In total, 27/32 (84%) of patients with very high serum S100B serum were MRI-negative and 4/27 with high S100B were CT+. Five MRI+ patients had elevated S100B. S100B sensitivity 100% with specificity of 81%.
Marchi et al., 2013	Prospective Cohort	15 athletes	3 T magnet; DTI	Serum S100B in college football players with MRI.	Serum S100B; MRI; neurocognitive tests	Before and after sports season	S100B post-game was elevated (0.51 ± 0.05 ng/mL) compared to baseline. Higher number of impacts correlated with increased S100B (*p* = 0.03). High S100B auto-antibodies correlated (*r* = 0.58, *p* = 0.07) with errors in impulse control
Oh et al., 2007	Prospective Cohort	101 TBI	unspecified	Serum S100B in patients admitted to EDt for TBI with CT/MRI.	Serum S100B; CT; MRI	On admission	Healthy controls had serum S100B 0.080 ug/L (0.049–0.094) compared to 0.150 ug/L (0.088–0.358) in acute TBI. 66/101 CT/MRI+ patients had higher S100B compared to CT/MRI-negative (*p* = 0.028). AT cutoff of 0.105 ug/L, sensitivity 84.8% and specificity 74.3% for detecting acute TBI.
Romner et al., 2000	Prospective Cohort	278 TBI	unspecified	Neurotrauma patients were evaluated for S100B levels on admission and compared with pathological findings on CT scan.	Serum S100B; CT; MRI	On admission	108/278 (39%) had elevated serum S100 and 25 (9%) were CT+. Serum S100B was higher in severe compared to mild-to-moderate TBI (*p* < 0.001). S100B was higher in those with intracranial pathology (*p* < 0.01). Sensitivity for CT/MRI+ was 92%, specificity was 66%.
Thelin et al., 2014	Retrospective Cohort	199 TBI	T1/T2; FLAIR; GRE	An analysis of serum increases in S100B levels post-TBI in addition to pathological imaging.	Serum S100B; CT; MRI	Three samples, with 1/3 taken > 48 h after injury	Secondary increases in S100B with a cutoff of 0.05 ug/L had sensitivity 80% and specificity 89%, while cutoff of 0.5 ug/L has sensitivity 16% and specificity 98% for imaging findings of TBI.
***GFAP, UCH-L1***							
**Author and Year**	**Study Type**	***N***	**MRI Acquisition Parameters**	**Description**	**Outcome Measures**	**Timing of Serum Sampling**	**Results**
Gill et al., 2018	Prospective Cohort	277 mTBI	T1/T2; FLAIR; 3D-T1; DTI	Plasma biomarkers were correlated with MRI data.	Plasma GFAP; tau; NFL; UCH-L1	Within 48 h of injury	mTBI had higher plasma GFAP, tau, and NF-L (*p* < 0.01). Patients with MRI findings had significantly higher concentrations of plasma GFAP, tau, and NF-L compared to MRI- and CT- mTBI patients (*p* < 0.05).
Kou et al., 2013	Prospective Cohort	Nine mTBI	3 T magnet; T1/T2; GRE; FLAIR; DTI	Feasibility testing of the utilization of both biomarkers and MRI to detect mTBI.	Serum UCH-L1 and GFAP levels; MRI data	Within 6 h of injury, and q6 h until 24 h post-injury	UCH-L1 (4.9-fold) and GFAP (10.6-fold) were elevated on admission in comparison to lab reference values. Patients with intracranial hemorrhages had higher GFAP compared to non-hemorrhage (*p* = 0.002). GFAP/UCH-L1 did not associate with MRI findings.
McMahon et al., 2015	Prospective Cohort	215 TBI	unspecified	Plasma GFAP-BDPs were used to predict CT/MRI+ TBI.	Plasma GFAP-BDPs; MRI	Within 24 h of injury	In total, 35% had evidence of TBI on MRI (*n* = 21). On admission, MRI+ patients had significantly higher GFAP-BDPs (1.3 ± 1.8 ng/m; *p* = 0.001 L) than MRI− (0.28 ± 0.57). Plasma concentrations of GFAP-BDPs predicted evidence of MRI pathology (OR 2.7; 95% CI 1.2–5.7).
Posti et al., 2017	Prospective Cohort	94 mTBI	3 T magnet; T1/T2; FLAIR; DTI; 3D-T1	Plasma GFAP, UCH-L1 in TBI were compared to orthopedic trauma.	Serum GFAP and UCH-L1; CT/MRI	On days 1,2,3,7 post-admission	None in the mTBI group showed signs of TBI on MRI. GFAP was initially higher in acute orthopedic trauma compared to acute CT−/MRI− mTBI (*p* = 0.026) with no difference days later. No difference in UCH-L1.
Yue et al., 2019	Prospective Cohort	450 TBI, 122 orthopedic and 207 healthy controls	T1/T2; FLAIR; GRE	Patients with negative initial CT, with MRI at 7–18 days, vs. orthopedic trauma controls and healthy controls.	Plasma GFAP and MRI	Within 24 h of injury	CT−/MRI+ (414.5 pg/mL; *p* < 0.001) patients had the highest plasma GFAP, compared to CT−/MRI− (74.0), orthopedic trauma (13.1) and healthy controls (8.0). AUC for discriminating MRI+ in CT− population was 0.852. GFAP was notably elevated for DAI on MRI, compared to other types of intracranial pathology.
***Tau***							
**Author and Year**	**Study Type**	***N***	**MRI Acquisition Parameters**	**Description**	**Outcome Measures**	**Timing of Serum Sampling**	**Results**
Hirad et al., 2019	Retrospective Cohort	29 mTBI	3 T magnet; 3D-T1; DTI	NCAA contact sport athletes with mTBI were monitored for concussion with plasma sampling and cognitive testing pre-/post-season.	White matter structural integrity using FA pre- and post-season, vs. age-matched controls, correlation between plasma tau and midbrain FA	Before and after sports season; mTBI patients had venipuncture within 72 h of injury	FA reduced in the right midbrain in concussed athletes compared to controls. Of 13/29 mTBI patients with blood samples, Tau was inversely related to midbrain FA (*r* = −0.60, *p* = 0.033).
Tomita et al., 2019	Prospective Observational	40 TBI	T2 FLAIR; DWI	TBI patients with acute symptoms < 6 h and patients with MRI evidence of DAI on T2WI/DWI regardless of symptoms.	Serum tau in DAI vs. non-DAI groups; sensitivity and specificity of Tau for DAI	Within 6 h of injury	All patients had high intensity areas on MRI within corpus callosum, brainstem and cerebrum with T2WI/DWI. Tau was higher in DAI (25.3 pg/mL; 0–99.1) vs. non-DAI (0.0 pg/mL; 0–44.4). At cutoff 1.5 pg/mL, sensitivity 74.1% and specificity 69.2% for DAI.
***NF-L***							
**Author and Year**	**Study Type**	***N***	**MRI Acquisition Parameters**	**Description**	**Outcome Measures**	**Timing of Serum Sampling**	**Results**
Ljungqvist et al., 2017	Prospective Cohort	Nine TBI	1.5 T; T1/T2; DTI	Serum NF-L levels in severe TBI patients were correlated with MRI and health outcomes.	Serum NF-L; MRI; GOSE	Within 24 h	All patients had DAI on MRI. NF-L ranged from 87.5–851.6 pg/mL. Severely disabled patients (GOSE 3–4) had higher serum NF-L (411 ± 263) than moderate (GOSE 5–6; 277 ± 80; *p* > 0.05)
Al Nimer et al., 2015	Prospective Cohort	182 TBI	echo-planar diffusion; FLAIR; GRE; T1/T2	Serum and CSF-NF-L levels were assessed in relation to imaging and outcomes.	Serum NF-L; CT; MRI; GCS; mortality	Sampled twice daily	In total, 159/182 patients survived, outcome data were available for 73% (116/159), GCS scores over time were significantly correlated with serum NF-L (*p* = 0.006) and weakly to CSF-NF-L (*p* > 0.05). Midline shift on MRI correlated with NF-L serum levels (*p* = 0.012).
Sandsmark et al., 2019	Prospective Cohort	30 controls, 56 TBI	1.5 T; T1/T2; SWI; DTI; FLAIR; GRE	Presence of traumatic hemorrhage or traumatic vascular injury correlated with serum NF-H.	Serum NF-H; MRI; GOS-E	Within 48 h of injury	Plasma NF-H was increased in both the traumatic hemorrhage and traumatic vascular injury groups compared to the MRI-negative group ≤ 48 h from injury and continued to stay increased in traumatic vascular injury cohort even 48 h after injury.
***Alpha-Synuclein***							
**Author and Year**	**Study Type**	***N***	**Age and Sex**	**Description**	**Outcome Measures**	**Timing of Serum Sampling**	**Results**
Ye et al., 2019	Prospective cohort	52	FLAIR; SWI; 3D-T1	Comparison of serum a-synuclein levels and brain connectivity in mTBI patients.	Serum a-synuclein; fMRI; PCS	On admission	All patients were CT-negative. Low levels of a-synuclein were associated with more severe PCS symptoms (*p* = 0.013) and depression (*p* = 0.022). Patients with decreased connectivity on fMRI had higher a-synuclein levels (*p* < 0.05).
***AMPAR***							
**Author and Year**	**Study Type**	***N***	**MRI Acquisition Parameters**	**Description**	**Outcome Measures**	**Timing of Serum Sampling**	**Results**
Dambinova et al., 2013	Prospective cohort	33 Concussion	1.5 T magnet; T1/T2 FLAIR	AMPAR peptide was measured in healthy controls and athletes with concussion to study its feasibility as a biomarker for TBI.	Serum AMPAR; MRI	On admission and again within 6 months of injury	AMPAR levels in controls were 0.05–0.40 ng/mL and 1.0–8.5 ng/mL in concussed athletes. AMPAR as a biomarker had a sensitivity of 91% and specificity 92% with 0.4 ng/mL cuff-off. Athletes having experienced multiple concussions had increased AMPAR levels (2.0–12.0 ng/mL) which was associated with MRI findings, though minor.

Caption: AMPAR = a-amitio-3-hydroxy-5-methyl-4-isoxazolepropionic acid receptor; CSF = cerebrospinal fluid; CT = computed tomography; DAI = diffuse axonal injury; DWI = diffusion-weighted imaging; ED = emergency department; FA = fractional anisotropy; GFAP = glial fibrillary acidic protein; GFAP-BDP = glial fibrillary acidic protein breakdown products; GOS = Glasgow Outcome Scale; GOSE = Glasgow Outcome Scale-Extended; MRI = magnetic resonance imaging; NCAA = National Collegiate Athletic Association; NF-L = neurofilament light chain; PCS = post-concussional symptoms; T2WI = T2-weighted imaging; TBI = traumatic brain injury; UCH-L1 = ubiquitin carboxyl-terminal hydrolase L1; GRE = gradient echo; DTI = diffusion tensor imaging; 3D-T1 = 3-dimensional T1; DTI = Diffusion Tractography Imaging; FLAIR = Fluid Attenuated Infusion Recovery.
